# Starch-based intelligent indicator membranes with triple antioxidant activity for fruit preservation via self-assembled berberine-oligomeric proanthocyanidins nanoparticles encapsulating tea tree oil

**DOI:** 10.1016/j.fochx.2026.104224

**Published:** 2026-07-20

**Authors:** Kang Zhang, Hefeng Zhu, Panliang Zhang, Yixuan He, Yixiu Wang

**Affiliations:** a Department of Chemistry and Chemical Engineering, Hunan Institute of Science and Technology, Yueyang 414006, China; b Department of Hepatic Surgery, Fudan University Shanghai Cancer Center, Shanghai 200032, China

**Keywords:** Packaging membrane, Triple antioxidant activity, Intelligent indicator, Self-assembled nanoparticles, Slow release

## Abstract

A multifunctional starch-based composite membrane was developed by incorporating self-assembled berberine-oligomeric procyanidins nanoparticles (SBO) loaded with tea tree oil (TTO) into a thermoplastic starch (TPS) matrix. Characterization indicated successful SBO formation and TTO loading (up to 62.36 mg/g), which followed a monolayer adsorption mechanism with pseudo-first-order kinetics. The incorporation of SBO@TTO significantly improved tensile strength and gas barrier properties of the membranes. The composite membranes exhibited excellent pH-responsive color changes and enhanced antioxidant and antibacterial activities. TTO release was sustained and diffusion-controlled, and the membranes exhibited satisfactory in vitro cytocompatibility. TPS/4%SBO@TTO membrane effectively reduced weight loss and softening of cherry tomatoes, showing better performance than air storage and comparable performance to a commercial polyethylene membrane. These results demonstrate the strong application potential of TPS/SBO@TTO membranes as active and intelligent food packaging. The direct migration and preservation performances should be further validated under various storage conditions and for different food products.

## Highlights

• Self-assembled nanoparticles (SBO) are used to load tea tree oil (TTO).
• The starch/SBO@TTO composite film exhibits distinct pH-responsive color changes.
• The starch/SBO@TTO composite film shows triple antioxidant activity.
• The TTO is released from the film via a sustained, diffusion-controlled mechanism.
• The TPS/4%SBO@TTO membrane significantly extends the shelf life of cherry tomatoes.

## Graphical abstract

**Figure:**
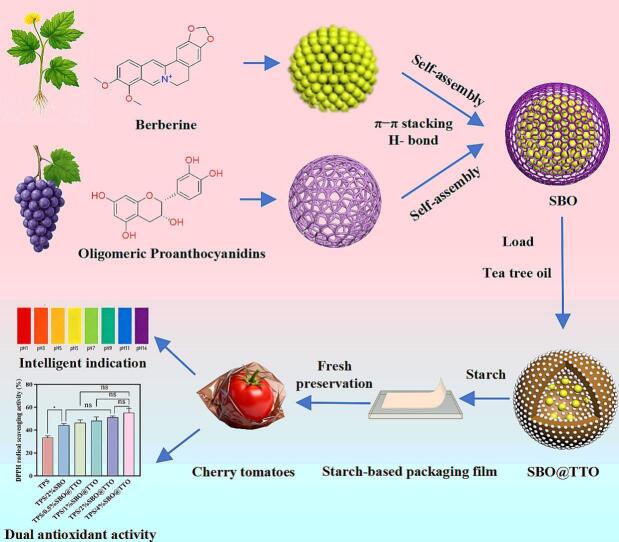

## 1 Introduction

Petroleum-based packaging materials pose significant environmental challenges owing to their poor biodegradability, which leads persistent pollution (Kumari et al., 2023). Furthermore, their limited functional properties often result in short food shelf-life and substantial economic losses (Haridevamuthu et al., 2024). In this context, starch has emerged as a promising alternative material owing to its renewability, biocompatibility, and inherent film-forming capacity (Dang et al., 2025). However, the practical application of pure starch films is limited by several inherent drawbacks, including inferior mechanical strength, high hygroscopicity, and a lack of active functionalities such as antioxidant and antimicrobial activities (Yudhistira et al., 2024). A strategic approach to overcoming these limitations involves incorporating of functional nanoparticles into the starch matrix (Dong et al., 2024). The development of starch-based nanocomposites, particularly those integrating nanoparticles with inherent bioactive properties, is an effective pathway for enhancing material performance while introducing valuable preservation capabilities, thereby addressing the critical needs for sustainable and active food packaging (Dhiman et al., 2025).

Self-assembled nanoparticles, formed through the spontaneous organization of molecular building blocks via non-covalent interactions, constitute a class of advanced nanomaterials with tunable structures and functionalities for applications in biomedical, electronic, and materials sciences (Král et al., 2024; Xu et al., 2025). Fu et al. (2024) developed a rabies vaccine candidate based on self-assembled nanoparticles derived from a ferritin architecture. This vaccine elicited robust, rapid, and durable immune responses, conferring complete protection against the live rabies virus in female mice after a single immunization. Tian et al. (2022) reported the fabrication of self-assembled particles composed of berberine and curcumin, which exhibited superior physicochemical stability and synergistic antimicrobial efficacy, while remaining non-toxic to plant cells.

Among the various bioactive compounds, berberine (BBR), a quaternary ammonium alkaloid, and oligomeric proanthocyanidins (OPCs), potent natural antioxidants, have attracted significant attention (Wen et al., 2025; Xu et al., 2024). BBR exhibits remarkable antibacterial and anti-inflammatory properties, whereas OPCs are highly effective scavengers of free radicals and inhibitors of lipid peroxidation (Gu et al., 2025; Xu et al., 2024). A recent study reported that self-assembled berberine-oligomeric proanthocyanidins nanoparticles (SBO) is proposed to occur via electrostatic and π-π stacking interactions (Huang et al., 2025). This self-assembly strategy enhances the stability and bioavailability of the individual components, and creates a novel platform for encapsulating hydrophobic bioactive substances. Tea tree oil (TTO), known for its excellent broad-spectrum antibacterial and antioxidant properties, is an ideal candidate for encapsulation (Li, Jiang, et al., 2024). However, its high volatility and poor water solubility severely limit its direct application in hydrophilic polymer matrices such as starch (Zhang et al., 2025).

Despite the promising properties of SBO and TTO, their combined application in starch-based intelligent packaging systems has not been systematically investigated. In particular, limited information is available on the use of SBO as carriers for TTO delivery, and their influence on the structural, functional, and preservation performance of starch-based packaging materials. The encapsulation of TTO within SBO has been proposed as a strategy to improve its stability, compatibility with the starch matrix, and controlled-release behavior. This study aimed to develop TTO-loaded SBO, incorporate them into thermoplastic starch membranes, and systematically evaluate their structural, functional, intelligent, and food-preservation properties.

## 2 Experimental

### 2.1 Materials and chemicals

Industrial-grade corn starch (Baoji Starch Co., Ltd., China) containing 14% moisture and exhibiting an average viscosity molecular weight of 1.55 × 10^7^ g/mol was used. Analytical-grade BBR, OPCs, gellan gum, glycerol, ethanol, calcium oxide, 2,2-diphenyl-1-picrylhydrazyl (DPPH), and deoxidizer were purchased from Macklin Biochemical Technology Co., Ltd. (Shanghai, China). TTO, composed of d-limonene (23.1%), p-cymene (17.0%), γ-terpinene (6.4%), 1,8-cineole (3.9%), and terpinen-4-ol (2.7%), was purchased from Macklin Biochemical Technology Co., Ltd. (Shanghai, China). Fresh cherry tomatoes were purchased from a fruit market in Yueyang, China.

### 2.2 Preparation of self-assembled nanoparticles

BBR (6 g) and OPCs (6 g) were dissolved in ethanol (1 L) and deionized water (1 L), respectively. The two solutions were mixed in a dialysis bag (MWCO 3500) at a mass ratio of 4: 1, and maintained at room temperature for 24 h. The mixture was centrifugated and freeze-dried (Huang et al., 2025). The product was designated as SBO. Ethanol and deionized water were selected as solvents instead of dimethyl sulfoxide to improve compatibility with food-packaging applications owing to their lower toxicity, easier removal during processing, and suitability for subsequent TTO loading.

### 2.3 Loading of TTO in SBO

SBO was immersed in a 1 g/L TTO/ethanol solution, and was sonicated for 2 h. The obtained suspension was subsequently filtered, and the remaining residue was dried at 25 °C. The final product was labeled as SBO@TTO. The loading capacity of TTO in SBO (*Q*, mg/g) was calculated using Eq. (1).

(1) $$Q\;\left(\mathrm{mg}/g\right)=\frac{m_{2}-m_{1}}{m_{1}}\times 1000$$

where *m*_1_ (g) and *m*_2_ (g) are the weights of the SBO before and after loading TTO, respectively.

Standard solutions of TTO/ethanol (50–500 mg/L) were prepared, and the absorbance of the filtrate was measured at 287 nm using a Specord S600 U*V*–Vis spectrophotometer (Jena, Germany). SBO (10 mg) and 20 mL TTO/ethanol solution (20 mg/L) were mixed, and left in the dark for different time periods (10, 20, 30, 60, 90, 120, 240, 480, and 720 min). The filtrate was separated by filtration, and the absorbance was analyzed using a UV–Vis spectrophotometer. The adsorption capacity of SBO for TTO at any time *t* (*Q*_t_, mg/g) was determined using Eq. (2).

(2) $$Q_{t}\;\left(\mathrm{mg}/g\right)=\frac{\left(C_{0}-C_{t}\right)\times V}{m}$$

where *m*, *C*_0_, *C*_t_, and *V* represent the weight of SBO (g), initial concentration of the TTO/ethanol solution, final concentration of the TTO/ethanol solution, and volume of the TTO/ethanol solution, respectively.

The adsorption isotherm of TTO in SBO was determined according to ASTM D3860–98. SBO (10 mg) and TTO/ethanol solutions of 20 mL at different concentrations (10, 20, 50, 75, 100, 150, 200, and 300 mg/L) were mixed, and incubated in the dark for 12 h. The suspension was then filtered, and the absorbance of the resulting filtrate was determined using a U*V*–Vis spectrophotometer at 287 nm. The adsorption capacity of SBO for TTO at equilibrium (*Q*_e_, mg/g) was determined using Eq. (3).

(3) $$Q_{e}\;\left(\mathrm{mg}/g\right)=\frac{\left(C_{0}-C_{e}\right)\times V}{m}$$

where *C*_e_ is the equilibrium concentration of the TTO/ethanol solution.

### 2.4 Preparation of the starch-based intelligent indicator membranes

Distilled water, glycerol, gellan gum, corn starch, and SBO@TTO were added to a three-necked flask. Glycerol and gellan gum were used as the plasticizer and reinforcing agent, respectively. The corn starch and gellan gum mixture, in an 8: 2 weight ratio, constituted 4% of the total weight. Glycerol comprised 20% of the combined weight of corn starch and gellan gum, whereas SBO@TTO was added at 0%, 0.5%, 1%, 2%, or 4% relative to the weight of the corn starch and gellan gum mixture. The mixture was then heated at 95 °C for 60 min, and the resulting starch paste was dried at 25 °C for 3 days. The starch membranes were labeled as TPS, TPS/0.5%SBO@TTO, TPS/1%SBO@TTO, TPS/2%SBO@TTO, and TPS/4%SBO@TTO based on the SBO@TTO fractions. A starch/gellan gum membrane with 2 wt% SBO (TPS/2%SBO) was prepared as a control using the same procedure.

### 2.5 Structural characterization of SBO, SBO@TTO, and starch membranes

The functional groups of the SBO, SBO@TTO, and starch membranes were analyzed using an FTS 3000 FTIR spectrometer (FTIR, Hercules, USA) following ASTM E1252 over a wavenumber range of 500 cm^−1^ to 4000 cm^−1^ with a resolution of 4 cm^−1^. ^1^H nuclear magnetic resonance (NMR) spectra of the BBR, OPCs, and SBO were recorded using an AVANCE III HD 400 MHz spectrometer (Bruker, Switzerland) at 25 °C using D_2_O as the solvent. The crystal structures of the SBO, SBO@TTO, and starch membranes were determined using an X'Pert Pro MPD X-ray diffraction (XRD) system (Philips, Netherlands) over a diffraction angle range of 5–80°, following ASTM E975. Elemental analyses of the SBO and SBO@TTO were conducted using a K-Alpha X-ray photoelectron spectrometer (XPS, Thermo Fisher Scientific, USA) with a monochromatic Al Kα X-ray source, following ISO 15472. The specific surface area, pore volume, and pore size distribution of the SBO were measured using a 3H-2000BET-A automatic surface area analyzer (BET, Beishide Instrument, China) following the ISO 9277 standard. Nitrogen was used as the adsorption gas. The morphologies and elemental compositions of the starch membranes were analyzed using a scanning electron microscopy (SEM) system equipped with an Ultim Max EDS attachment (Thermo Fisher, USA) at an acceleration voltage of 20 kV.

### 2.6 Performance testing of the starch membranes

The tensile properties of the starch membranes were measured using a YG 061–1500 electronic strength tester (Laizhou, China) according to the ASTM D882 standard. The initial length was set to 100 mm, and the stretching speed was 100 mm/min. The average thickness of the starch membranes was 0.2 mm, measured at five random positions using a digital micrometer. The tensile properties were measured in triplicate.

The transparency of the starch membranes was observed from photographs, and measured using a UV–Vis spectrophotometer over the wavelength range of 200–800 nm according to ASTM D1003. The morphologies of the starch membranes at different pH values were analyzed using photographs.

Water contact angle of the starch membranes were measured using an OCA25 contact angle meter (Dataphysics, Germany) according to ASTM D7334. The moisture content (*M*_*c*_) of the starch membranes was determined according to the ASTM D570 standard, and averaged three times.

The water vapor permeability (WVP, g·cm/(cm^2^·s·Pa)) of the starch membranes was measured three times using a C360M water vapor transmission rate tester (Languang, China) according to ASTM E96. The oxygen transmission rate (*OTR*, cm^3^·cm/(cm^2^·s·Pa)) of the starch membranes was determined using a GTT gas permeation meter (Brugger, Germany) according to the ASTM D1434 standard.

The DPPH scavenging activity of the starch membranes (*R*_DPPH_, %) was measured three times, and calculated using Eq. (4). One milliliter of starch-ethanol solution was combined with 4 mL of DPPH-ethanol solution and incubated in the dark for 60 min. The absorbance of the solution was measured using a UV–Vis spectrophotometry.

(4) $$R_{\text{DPPH}}=\frac{\left(A_{b}-A_{s}\right)}{A_{b}}\times 100$$

where *A*_b_ and *A*_s_ represent the absorbance of the DPPH solutions at 517 nm with and without starch membranes, respectively.

*Escherichia coli* (*E. coli*) and *Staphylococcus aureus* (*S. aureus*) were cultured in an SHP-150 digital biochemical incubator (Ronghua, China) at 37 °C for 12 h, and then diluted to one-tenth of their initial concentration. Subsequently, the starch membranes were separately co-cultured with *E. coli* and *S. aureus.* The antibacterial activity of the starch membranes was evaluated using the colony-forming unit (CFU) reduction method (Zhang et al., 2020). Membrane samples were incubated in nutrient broth containing 0.1–1 × 10^6^ CFU/mL of *E. coli* or *S. aureus* at 37 °C for 2 h under shaking at 150 rpm. Subsequently, aliquots of the bacterial suspensions were serially diluted, plated onto nutrient agar, and incubated for 24 h. The number of viable colonies was counted, and the antibacterial activity was expressed as the inhibition rate relative to the blank control. Antibacterial activity assays were performed in triplicate.

The starch membranes were sterilized, and placed in LO2 cell culture dishes for 24 h, with PBS as the control. LO2 cells were digested and seeded in 96-well plates at a density of 5 × 10^3^ cells/well. After 24 h of incubation, cell viability was evaluated using the CCK-8 assay. The cells were incubated with CCK-8 reagent for 1 h, and the optical density (OD) at 450 nm was measured using a microplate reader.

Following treatment with the starch membranes, LO2 cells were digested again, and 50 μL of the cell suspension was mixed with an equal volume of trypan blue stain. The mixture was loaded into a hemocytometer, and the cells were counted under a microscope within 10 min.

Cherry tomatoes were randomly assigned to treatment groups. The preservation experiment was conducted at 25 ± 1 °C and 50 ± 5% relative humidity for 18 days, with three independent replicates for each treatment. The appearance of the cherry tomatoes was observed at selected time points, and their weight loss rate (%) was calculated using Eq. (5). The hardness (N) of cherry tomatoes preserved with starch membranes was measured using a GY-4 fruit hardness tester (Puyan, China). Sugar content was quantified using a WYA-2S digital Abbe refractometer (Honglin, China), and surface pH was assessed after 18 days using a flat-surface electrode (Mettler-Toledo, Switzerland) on three randomly sampled tomatoes per package. The CIELab color parameters (*L*, *a*, and *b*) of both the tomato surface and packaging membrane were evaluated using a DS-100 color difference meter (Caipei, China). The *L* value represents the lightness (0 = black, 100 = white), the *a* value represents the red-green axis (positive = red, negative = green), and the *b* value represents the yellow-blue axis (positive = yellow, negative = blue). A trained sensory panel (*n* = 10) rated the appearance, odor, texture, taste, and overall liking of the cherry tomatoes on a nine-point hedonic scale, and the evaluation was performed in accordance with ethical requirements.

(5) $$\text{Weight loss rate}=\frac{\left(m_{3}-m_{4}\right)}{m_{3}}\times 100$$

where *m*_3_ (g) and *m*_4_ (g) represented the weight of the cherry tomatoes before and after storage, respectively.

### 2.7 Release kinetics of TTO from the TPS/4%SBO@TTO membrane

To simulate controlled release, a TPS/4%SBO@TTO membrane with a size of 3 × 3 cm^2^ was sealed in dialysis tubing (12–14 kDa) and immersed in 100 mL of ethanol. Aliquots (3 mL) were collected at set intervals, and their absorbance at 287 nm was measured using a UV–Vis spectrophotometer. The cumulative release of TTO was modeled using the Avrami (Eq. (6)) and Higuchi (EEq. (7)) equations.

(6) $$R\left(\%\right)=\frac{M_{t}}{M_{\infty }}=1-\exp\left(-{\left(\mathrm{kt}\right)}^{n}\right)$$

where *R* is the release ratio (%), *M*_t_ and *M*_∞_ are the release amounts (g) at time *t* and at equilibrium, respectively, *k* is the rate constant (s^−1^), *t* is the time (s), and *n* is a mechanism parameter.

(7) $$Q/A=2C_{0}\sqrt{Dt/\pi }$$

where *Q* is the released amount (mg), *C*_0_ is the initial concentration (mg/mL), *A* is the diffusion area (cm^2^), *D* is the apparent diffusion coefficient (cm^2^/min), and *t* is measured in minutes.

### 2.8 Statistic analysis

Statistical analyses were performed using SPSS software (version 26.0, IBM Corp., Armonk, NY, USA). All experiments were conducted in triplicate (*n* = 3) unless otherwise specified, and the results are expressed as the mean ± standard deviation (SD). One-way analysis of variance (ANOVA) was used to evaluate the statistical significance of the differences between multiple experimental groups. When a significant F-test result was obtained (*P* < 0.05), post-hoc multiple comparisons were conducted using Tukey's honestly significant difference (HSD) test to identify specific group differences. For pairwise comparisons between two groups, Student's *t*-test was applied. All statistical tests were two-tailed, and differences were considered significant at *P* < 0.05. Significance levels are indicated in the figures as follows: P < 0.05 (*), *P* < 0.01 (**), and *P* < 0.001 (***).

## 3 Results and discussion

### 3.1 Structural characterization of SBO and SBO@TTO

**Fig. 1:**
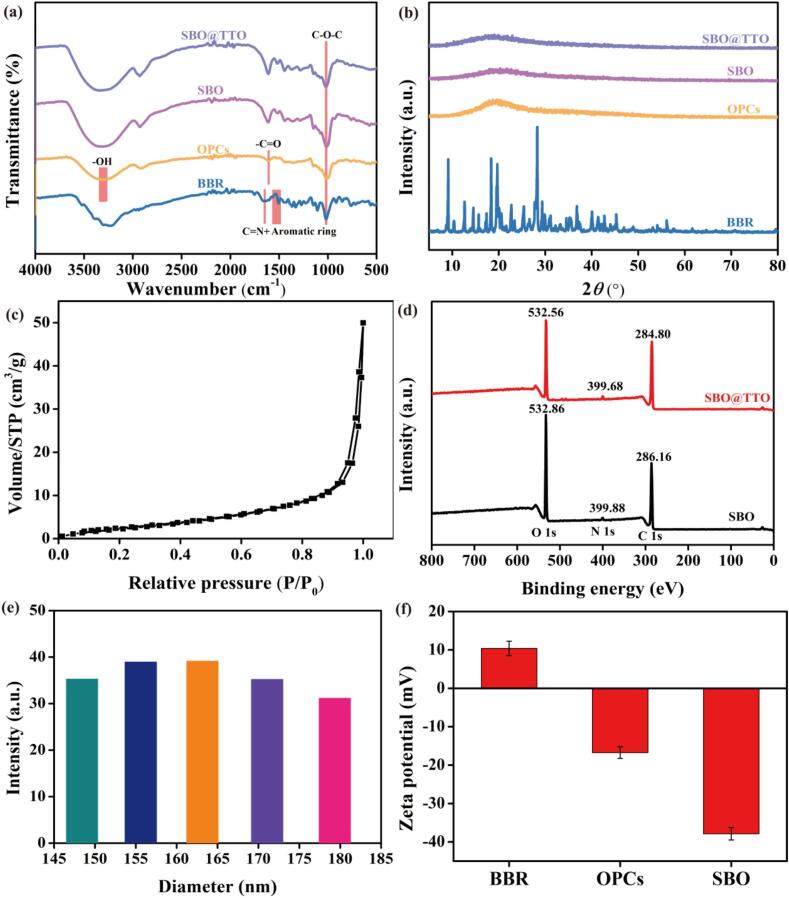
Structural characterization of SBO and SBO@TTO, (a) FTIR, (b) XRD, (c) BET, (d) XPS, (e) diameter, and (f) zeta potential.

FTIR spectra of the SBO and their corresponding raw materials are shown in Fig. 1(a). In the BBR spectrum, the peak at 1648 cm^−1^ was assigned to the –C=N+ stretching vibration, whereas the peak at 1510 cm^−1^ corresponds to the vibration of the aromatic ring skeleton. OPCs exhibit distinct peaks at 3310 cm^−1^ (–OH), 1608 cm^−1^ (–C=O), and 1001 cm^−1^ (–C–O). Compared to the spectra for BBR and OPCs, no new peaks are observed in the SBO spectrum; however, the –C–O stretching vibration shifts from 1001 cm^−1^ to 1010 cm^−1^. This shift is consistent with the formation of non-covalent interactions between BBR and OPCs during self-assembly, likely through hydrogen bonding and π-π stacking (Huang et al., 2025). No significant differences are observed between the SBO@TTO and SBO spectra.

The XRD patterns of the SBO and their corresponding raw materials are shown in Fig. 1(b). The BBR pattern exhibits distinct diffraction peaks at 9.1°, 20.0°, and 28.1°, which are indexed to the (001), (012), and (114) crystal planes, respectively (Zhang et al., 2025). In contrast, both OPCs and SBO show broad halo patterns, indicative of amorphous structures. Upon self-assembly with SBO, the original crystalline lattice of BBR is disrupted, which is indicative of the formation of a disordered nanoscale architecture (Tian et al., 2022).

The N_2_ adsorption-desorption isotherms for the SBO are presented in Fig. 1(c). The specific surface area and pore volume of the SBO are 9.80 m^2^/g and 0.08 cm^3^/g, respectively. The pore size of the SBO is 3.76 nm, implying a mesoporous structure, indicating that it may effectively load TTO.

The XPS profiles of SBO and SBO@TTO are presented in Fig. 1(d). The SBO spectrum shows the C 1 s, N 1 s, and O 1 s peaks are observed at 286.16 eV, 399.88 eV, and 532.86 eV, respectively. Upon loading with TTO, the peaks shift to lower binding energies: C 1 s to 284.80 eV, N 1 s to 399.68 eV, and O 1 s to 532.56 eV. The consistent reduction in the binding energy may indicate an increase in the electron density around these atoms, probably resulting from the non-covalent interactions triggered by TTO incorporation (He, Fan, et al., 2025).

Fig. 1(e) shows that the SBO has a relatively narrow size distribution with an average hydrodynamic diameter of 162.98 ± 10.61 nm (polydispersity index, PDI = 0.042), indicating a homogeneous population of nanoparticles. As shown in Fig. 1(f), the zeta potential of the SBO was determined to be −37.88 ± 1.62 mV, suggesting excellent stability due to the strong electrostatic repulsion between negatively charged particles (Huang et al., 2025). This highly negative surface charge is probably attributed to the abundant hydroxyl groups of the OPCs, which are hypothesized to remain exposed on the nanoparticle surface after self-assembly with BBR. The favorable size and surface charge characteristics of SBO are expected to facilitate their uniform dispersion within the starch matrix and contribute to the enhanced functional properties of the composite membranes.

^1^H NMR spectra of BBR, OPCs, and SBO are shown in Fig. S1. The characteristic proton signals of BBR are observed at *δ* positions of 3.302, 3.663, 3.765, 4.111, 4.863, and 5.100 ppm, consistent with previous reports (Huang et al., 2025). The OPCs spectrum exhibits proton signals at 3.331, 4.621, 4.860, 4.869, and 4.898 ppm, corresponding to the protons of the flavonoid units. In the SBO spectrum, significant changes in the chemical shifts are observed compared to those of the individual components. The signal at *δ* 3.302 in the SBO spectrum shows a slight shift compared to that of BBR, whereas the OPCs signal at *δ* 4.621 shifts to *δ* 4.589 in the SBO spectrum. The NMR results, including these chemical shift perturbations, is consistent with the interpretation that the self-assembly of BBR and OPCs into SBO is likely driven by non-covalent interactions (Huang et al., 2025). Specifically, the upfield shifts of the aromatic protons are characteristic of π-π stacking interactions between the aromatic rings of BBR and the phenolic rings of OPCs. Additionally, the changes in the chemical shifts of the protons adjacent to the charged moieties may reflect the involvement of electrostatic interactions between the positively charged quaternary ammonium group of BBR and the negatively charged phenolic hydroxyl groups of OPCs. The broadening and disappearance of certain signals could also be associated with the formation of hydrogen bonds between the hydroxyl groups of OPCs and the electronegative atoms of BBR. While these spectral features are consistent with the proposed interactions, direct confirmation would require complementary techniques such as molecular dynamics simulations or X-ray crystallography.

### 3.2 Adsorption kinetics and isotherm of SBO for TTO

**Fig. 2:**
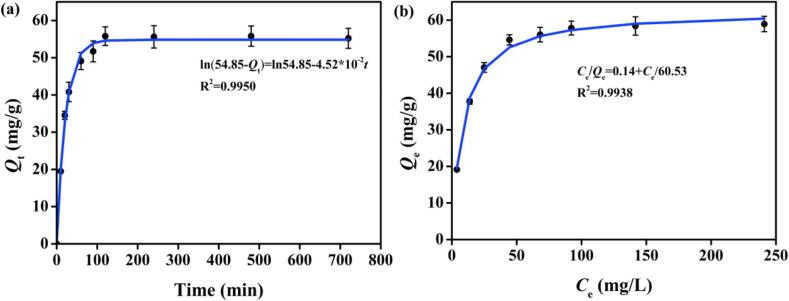
(a) Adsorption kinetics and (b) isotherm of TTO adsorbed on the SBO.

The adsorption kinetics and isotherms of TTO loaded onto the SBO are presented in Fig. 2. As shown in Fig. 2(a), the adsorption kinetics of TTO onto the SBO are best described by a pseudo-first-order kinetic model. The fitted equation is expressed as ln (54.85 − *Q*_t_) = ln 54.85–4.52 × 10^−2^ × *t*, with a high correlation coefficient (R^2^ = 0.9950), indicating that this model accurately describes the adsorption kinetics. The close agreement between the experimental data and the model suggests that the adsorption of TTO onto the SBO follows a monolayer adsorption mechanism under the given conditions (Jiang et al., 2025).

Furthermore, the adsorption isotherm, illustrated in Fig. 2(b), fits well with the Langmuir model, supporting the hypothesis of monolayer adsorption onto a homogeneous surface (Liu et al., 2024). The linearized form of the isotherm was fitted as *C*_e_/*Q*_e_ = 0.14 + *C*_e_/60.53 (R^2^ = 0.9938). The excellent fit of the Langmuir model suggests that TTO adsorption occurs predominantly via monolayer coverage of relatively homogeneous adsorption sites on the SBO. The theoretical maximum adsorption capacity calculated using the Langmuir model is 60.53 mg/g. The experimentally determined loading capacity of TTO on the SBO is 62.36 ± 1.92 mg/g, which closely agrees with the theoretical value. This minor deviation may be attributed to experimental variations or interactions between the adsorbate molecules.

### 3.3 The structural characterization of the starch membranes

**Fig. 3:**
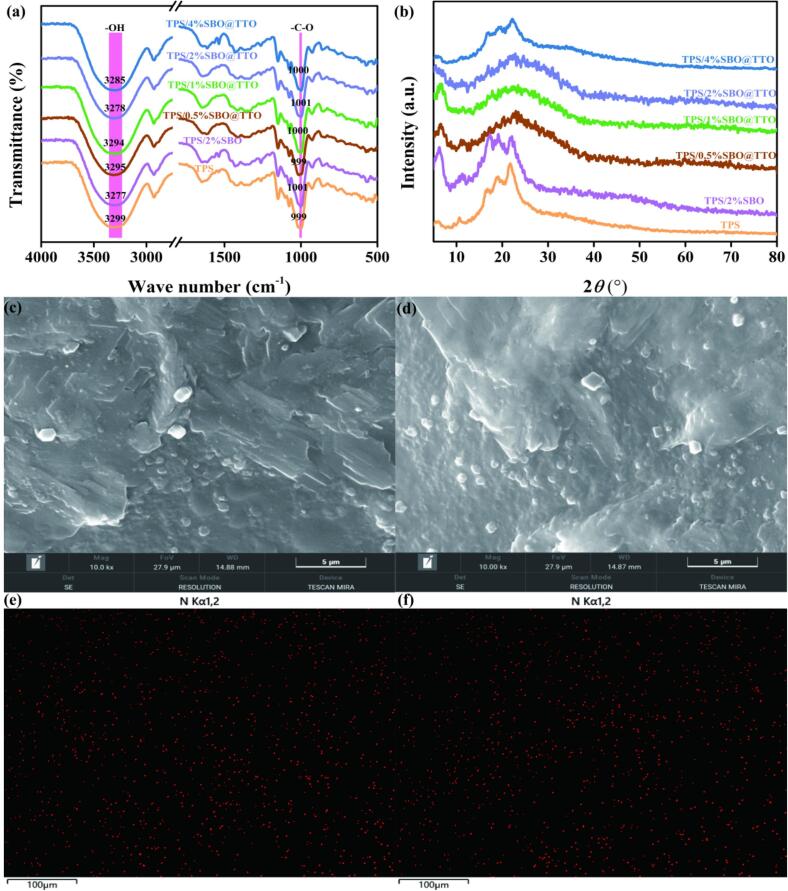
Structural characterization of the starch membranes, (a) FTIR, (b) XRD, (c) SEM image of TPS/2%SBO, (d) SEM image of TPS/4%SBO@TTO, (e) elemental mapping of TPS/2%SBO, and (f) elemental mapping of TPS/4%SBO@TTO.

The functional groups and intermolecular interactions of the starch membranes were determined using FTIR spectroscopy, as shown in Fig. 3(a). The peaks at 3299 cm^−1^ and 999 cm^−1^ in the FTIR spectrum of the starch membranes correspond to the stretching vibration absorption peaks of –OH and C—O in starch, respectively. In the FTIR spectra for the TPS/SBO and TPS/SBO@TTO membranes, the –OH stretching vibration absorption peaks are observed at lower wavenumbers, suggesting that the polar groups in SBO and SBO@TTO formed stronger hydrogen bond interactions with the –OH groups in starch than with those between starch molecules (Kong et al., 2024).

The XRD patterns of the starch membranes are shown in Fig. 3(b). The peaks at 14.7°, 17.0°, 18.6°, 19.8°, and 22.0° in the XRD pattern of the TPS membrane correspond to the characteristic diffraction peaks of B-type and V_h_-type starch crystals. The addition of SBO and SBO@TTO did not alter the starch crystal type. The crystallinities of the TPS, TPS/2%SBO, TPS/0.5%SBO@TTO, TPS/1%SBO@TTO, TPS/2%SBO@TTO, and TPS/4%SBO@TTO membranes are 23.0%, 36.5%, 32.0%, 32.4%, 34.7%, and 37.6%, respectively. These results suggest that SBO and SBO@TTO enhance starch crystallization, potentially by promoting heterogeneous nucleation and facilitating crystal growth (Son et al., 2025).

SEM images and elemental mapping of the TPS/2%SBO and TPS/4%SBO@TTO membranes are presented in Fig. 3(c–f). The SBO and SBO@TTO are evenly dispersed in the starch matrix, indicating their good compatibility (Zhang et al., 2025).

### 3.4 Morphology and intelligent indication of the starch membranes

**Fig. 4:**
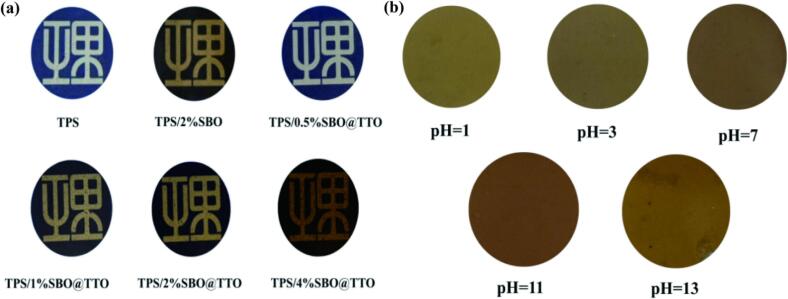
(a) Morphology and (b) intelligent indication photos of the starch membranes.

Photographs of the starch membrane morphologies are shown in Fig. 4(a). The TPS membrane is nearly colorless and transparent. Compared to the TPS membrane, the starch membranes containing SBO or SBO@TTO are reddish-brown and more opaque. The color of the starch/SBO@TTO membranes gradually deepens as the SBO@TTO content increases. This is supported by the UV–Vis analysis and color difference measurements, as shown in Fig. S2 and S3, respectively. This color change is attributed to the higher stacking degree of SBO@TTO and higher crystallinity of the starch/SBO@TTO membranes with increasing SBO@TTO loading. The transmittances of the TPS, TPS/2%SBO, TPS/0.5%SBO@TTO, TPS/1%SBO@TTO, TPS/2%SBO@TTO, and TPS/4%SBO@TTO membranes at 300 nm are 45.6%, 3.4%, 27.4%, 19.9%, 5.6%, and 1.8%, respectively. The TPS/4%SBO@TTO membrane with the highest UV-shielding is beneficial for reducing fruit deterioration due to UV absorption (Wei et al., 2024). In addition, the CIELab *a* and *b* values of the TPS/SBO@TTO membranes after 18 days cherry tomato preservation are higher, whereas the *L* value is lower. The higher *a* and *b* values may be attributable to the combined effects of pH variation and the accumulation of natural pigments from the fruit surface on the membrane.

Photographs showing the color changes in the TPS/4%SBO@TTO membrane at different pH values are presented in Fig. 4(b). At pH 3, the membrane exhibits a distinct pistac, which is attributed to the formation of flavylium cations from oligomeric procyanidins. At pH 7, the membrane appears brown owing to the combination of the bluish-violet hue of the oligomeric procyanidins and the yellow color contributed of BBR. Under alkaline conditions (pH 11), the membrane appears dark brown because of the progressive conversion of OPCs to quinone bases, pseudobases, or chalcone structures, resulting in a change in the conjugated system and a red shift in the absorption peaks. These pronounced color transitions are visually striking and highly useful as environmental indicators in practical applications. The clear and pH-dependent color response demonstrates the potential of the TPS/4%SBO@TTO membrane as an effective indicator for intelligent packaging, environmental monitoring, and biomedical sensing. The reversible and distinct color variations across a broad pH range offer great promise for real-time, visual detection of spoilage in food products, shifts in environmental conditions, and even physiological changes, without the need for complex instrumentation (Zheng et al., 2024). This functionality highlights the versatility and potential contribution of this material to sustainable and intelligent technologies.

### 3.5 Tensile properties of the starch membranes

**Fig. 5:**
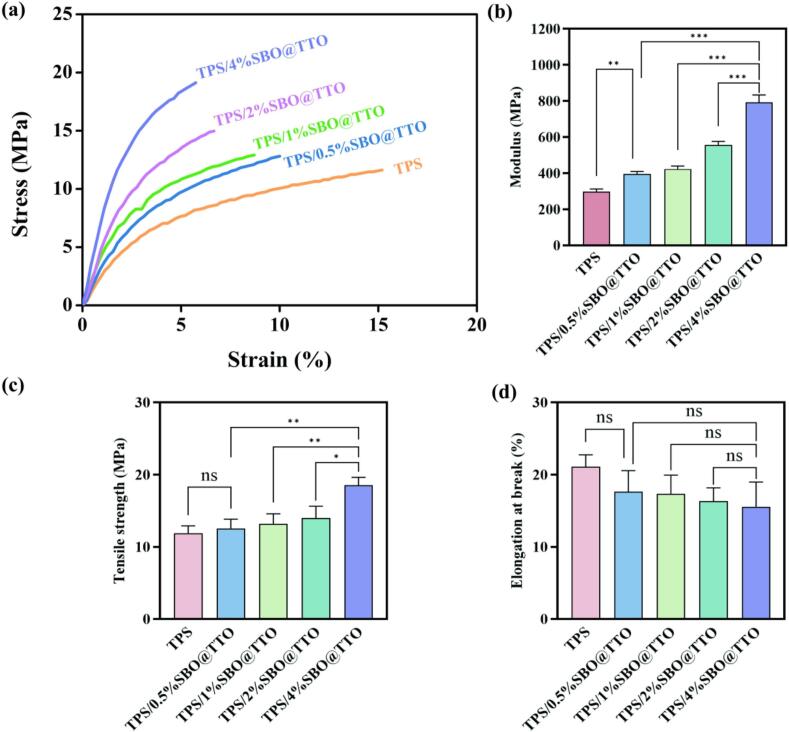
Tensile properties of the starch membranes, (a) stress-strain curves, (b) elastic modulus, (c) tensile strength, and (d) elongation at break. Asterisks indicate significant differences: *P < 0.05, **P < 0.01, ***P < 0.001.

The tensile properties of the starch membranes are shown in Fig. 5. As shown in Fig. 5(a), all the studied membranes exhibit ductile fracture behavior owing to the presence of glycerol. As illustrated in Fig. 5(b–d), the tensile strength and elastic modulus of the starch/SBO@TTO composite membranes increases with increasing SBO@TTO content, whereas the elongation at break gradually decreases. This may be due to the following reasons (Song et al., 2023). (1) SBO as a nucleating agent, can accelerate the nucleation and crystallization rate of starch, resulting in an increase in the crystallinity of the starch membranes (Fig. 3b), which eventually leads to an enhancement in the tensile strength and elastic modulus of the starch membranes. (2) SBO have strong interactions and good compatibility with starch molecular chains (Fig. 3(a, d)), which hinder chain movement and enable efficient stress transfer to the rigid nanoparticles.

In Fig. 5(b, c), the elastic modulus and tensile strength of the TPS/4%SBO@TTO membrane are 792.8 ± 41.0 MPa and 18.5 ± 1.1 MPa, respectively, which are significantly higher than those of the other TPS/SBO@TTO composite membranes (P < 0.05). In contrast, there were no significant differences in the elongation at break among the TPS/SBO@TTO composite membranes, as shown in Fig. 5(d) (*P* > 0.05). Considering the elastic modulus and tensile strength, the optimal SBO@TTO loading in the TPS/SBO@TTO composite membranes is 4 wt%.

### 3.6 Hydrophilicity and gas barrier performance of the starch membranes

**Fig. 6:**
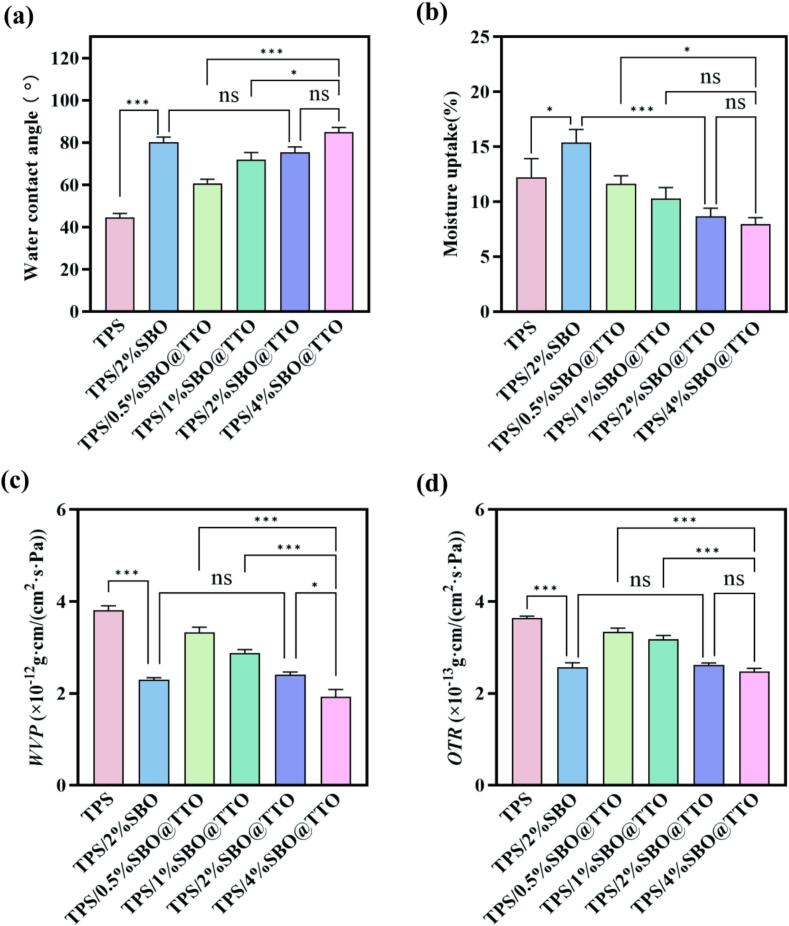
Hydrophilicity and gas barrier properties of the starch membranes, (a) water contact angle, (b) moisture uptake, (c) water vapor permeability, and (d) oxygen transmission rate. Asterisks indicate significant differences: *P < 0.05, **P < 0.01, ***P < 0.001.

Starch is a highly hydrophilic and hygroscopic material, which limits its application in packaging applications. The hydrophilicity of the starch membranes was determined from the water contact angle. As shown in Fig. 6(a), the water contact angles for the TPS, TPS/2%SBO, TPS/0.5%SBO@TTO, TPS/1%SBO@TTO, TPS/2%SBO@TTO, and TPS/4%SBO@TTO membranes are 44.6 ± 1.4°, 80.1 ± 1.8°, 60.7 ± 1.4°, 72.0 ± 2.4°, 75.3 ± 1.9°, and 85.0 ± 1.5°, respectively. The TPS/4%SBO@TTO membrane shows a significantly higher water contact angle than the TPS/1%SBO@TTO (*P* < 0.05) and TPS/0.5%SBO@TTO (P < 0.001) membranes. However, no significant difference is observed between TPS/4%SBO@TTO and TPS/2%SBO@TTO (*P* > 0.05), suggesting that further increasing the SBO@TTO content beyond 2 wt% does not result in a significantly enhancement of surface hydrophobicity. Notably, the TPS/2%SBO membrane also exhibits a significantly higher water contact angle than the TPS membrane (*P* < 0.001), confirming that the incorporation of SBO alone improved hydrophobicity. The enhanced hydrophobicity of the starch/SBO@TTO composite membranes is attributed to two main factors: (1) the hydrophobic nature of SBO@TTO, which reduces the overall hydrophilicity of the starch matrix, and (2) the rough surface topography imparted by the nanoparticles, which creates a lotus-leaf-like effect that enhances water repellency (Cao et al., 2024).

The moisture uptake of the starch membranes are shown in Fig. 6(b). The moisture uptake of the TPS, TPS/2%SBO, TPS/0.5%SBO@TTO, TPS/1%SBO@TTO, TPS/2%SBO@TTO, and TPS/4%SBO@TTO membranes are 12.2 ± 1.7%, 8.4 ± 1.1%, 11.6 ± 0.7%, 10.3 ± 1.0%, 8.7 ± 0.7%, and 7.9 ± 0.6%, respectively. The moisture uptake of the starch/SBO@TTO composite membranes decreases progressively as the SBO@TTO content increases, indicating a concentration-dependent effect. The reduced moisture uptake of the starch/SBO@TTO composite membranes can be attributed to three factors. First, the hydrophobic nature of SBO@TTO decreases the overall hydrophilic proportion of the starch matrix, reducing its affinity for water molecules. Second, the polar groups on SBO@TTO can form strong hydrogen bonds with the hydroxyl groups in starch, partially occupying the active sites and limiting its moisture absorption capacity (He, Li, et al., 2025). Third, the compact structure of the starch/SBO@TTO composite membranes, resulting from the well-dispersed nanoparticles and enhanced starch crystallization, creates a more tortuous diffusion path for water molecules, thereby hindering their penetration through the membrane (He et al., 2024). Post-hoc Tukey's HSD analysis reveals that the TPS/4%SBO@TTO membrane exhibits the lowest moisture uptake, which is significantly lower than that of the TPS/0.5%SBO@TTO membrane (*P* < 0.05), confirming that higher SBO@TTO loadings effectively reduce moisture absorption. However, no significant differences are observed between TPS/4%SBO@TTO and TPS/2%SBO@TTO (*P* > 0.05), or between TPS/4%SBO@TTO and TPS/1%SBO@TTO (P > 0.05), suggesting that the moisture uptake reduction plateaus at higher SBO@TTO concentrations. Notably, the TPS/2%SBO membrane exhibits significantly lower moisture uptake than the TPS/2%SBO@TTO membrane (*P* < 0.001), indicating that the incorporation of SBO alone is more effective in reducing moisture uptake than the equivalent amount of SBO@TTO. This suggests that the encapsulation of TTO within SBO may partially shield the polar groups of SBO, which are responsible for hydrogen bonding with starch hydroxyl groups, thereby slightly diminishing the moisture barrier effect. Additionally, the TPS/2%SBO membrane shows significantly lower moisture uptake than the TPS membrane (P < 0.05), confirming the beneficial effect of SBO incorporation on moisture resistance.

The gas barrier properties of packaging *materials*, such as *WVP* and *OTR*, play a crucial role in determining the shelf life of foods. The *WVP* values of the starch membranes are shown in Fig. 6(c). The *WVP* values of the TPS, TPS/2%SBO, TPS/0.5%SBO@TTO, TPS/1%SBO@TTO, TPS/2%SBO@TTO, and TPS/4%SBO@TTO membranes are 3.8 × 10^−12^ g·cm^2^/(cm·s·Pa), 2.3 × 10^−12^ g·cm^2^/(cm·s·Pa), 3.3 × 10^−12^ g·cm^2^/(cm·s·Pa), 2.9 × 10^−12^ g·cm^2^/(cm·s·Pa), 2.4 × 10^−12^ g·cm^2^/(cm·s·Pa), and 1.9 × 10^−12^ g·cm^2^/(cm·s·Pa), respectively. One-way ANOVA reveals significant differences in *WVP* among the starch membranes. As the addition amount of SBO@TTO increases, the *WVP* values of the starch/SBO@TTO composite membranes progressively decrease, indicating a concentration-dependent improvement in the water vapor barrier performance. Both SBO and dense structure of the membranes create a more convoluted path for water molecules travelling through the starch/SBO@TTO composite membranes (Dong et al., 2025). Post-hoc Tukey's HSD analysis reveals that the TPS/4%SBO@TTO membrane exhibits the lowest *WVP*, which is significantly lower than that of TPS/2%SBO@TTO (P < 0.05), TPS/1%SBO@TTO (*P* < 0.001), and TPS/0.5%SBO@TTO (P < 0.001). These results confirm that increasing SBO@TTO content significantly enhances the water vapor barrier properties of the starch-based membranes. Notably, no significant difference is observed between TPS/2%SBO and TPS/2%SBO@TTO (*P* > 0.05), suggesting that the incorporation of SBO alone provides a water vapor barrier enhancement comparable to that of SBO@TTO at the same concentration. This indicates that the SBO themselves play a dominant role in improving the barrier properties, likely by creating a tortuous path for water vapor diffusion, and the TTO encapsulated within SBO@TTO does not significantly compromise this effect. However, both TPS/2%SBO and TPS/2%SBO@TTO exhibit significantly lower *WVP* values than the TPS membrane (P < 0.001), confirming the substantial improvement in water vapor barrier performance achieved by nanoparticle incorporation.

The *OTR* values for the starch membranes are shown in Fig. 6(d). The *OTR* values of the TPS, TPS/2%SBO, TPS/0.5%SBO@TTO, TPS/1%SBO@TTO, TPS/2%SBO@TTO, and TPS/4%SBO@TTO membranes are 3.6 × 10^−13^ cm^3^·cm/(cm·s·Pa), 2.6 × 10^−13^ cm^3^·cm/(cm·s·Pa), 3.3 × 10^−13^ cm^3^·cm/(cm·s·Pa), 3.2 × 10^−13^ cm^3^·cm/(cm·s·Pa), 2.6 × 10^−13^ cm^3^·cm/(cm·s·Pa), and 2.5 × 10^−13^ cm^3^·cm/(cm·s·Pa), respectively. One-way ANOVA reveals significant differences in the *OTR* among the starch membranes. Starch-based membranes incorporating SBO or SBO@TTO exhibit lower *OTR* values compared to the TPS membranes. Furthermore, as the concentration of SBO@TTO increases, the *OTR* values of the starch/SBO@TTO composite membranes progressively decrease, indicating a concentration-dependent improvement in the oxygen barrier performance. The lower *OTR* values of the starch/SBO@TTO composite membranes are attributed to the longer diffusion path for oxygen molecules introduced by the well-dispersed SBO or SBO@TTO within the starch matrix (Dong et al., 2025). The dense and compact structure of the composite membranes, resulting from the strong interfacial interactions between the nanoparticles and starch matrix, further restricts oxygen penetration through the membrane. Post-hoc Tukey's HSD analysis reveals that the TPS/4%SBO@TTO membrane exhibits the lowest *OTR*, which is significantly lower than that of TPS/1%SBO@TTO (P < 0.001) and TPS/0.5%SBO@TTO (P < 0.001), confirming that higher SBO@TTO incorporation significantly enhances the oxygen barrier properties. However, no significant difference is observed between TPS/4%SBO@TTO and TPS/2%SBO@TTO (P > 0.05), suggesting that the oxygen barrier improvement plateaus when the SBO@TTO content increases above 2 wt%. Notably, no significant difference is observed between TPS/2%SBO and TPS/2%SBO@TTO (P > 0.05), indicating that the incorporation of SBO alone provides an oxygen barrier enhancement comparable to that of SBO@TTO at the same concentration. This suggests that the SBO themselves play a dominant role in improving the oxygen barrier performance, likely by creating a tortuous diffusion path for oxygen molecules, and the encapsulated TTO within SBO@TTO does not significantly degrade this effect.

The compact and dense structure of the starch/SBO@TTO composite membranes, resulting from strong interfacial interactions and enhanced crystallization, creates a tortuous diffusion path for oxygen and water vapor molecules. Similar reductions in *WVP* have been reported for starch membranes incorporating ZnO nanoparticles and essential oils (Sarhadi et al., 2024). Compared with these recently reported starch-based active membranes, the TPS/4%SBO@TTO membrane exhibits competitive *WVP* and *OTR* values, confirming the effectiveness of SBO@TTO incorporation in improving the barrier performance.

### 3.7 Antioxidant, antibacterial, and cytocompatibility evaluation of the starch membranes

**Fig. 7:**
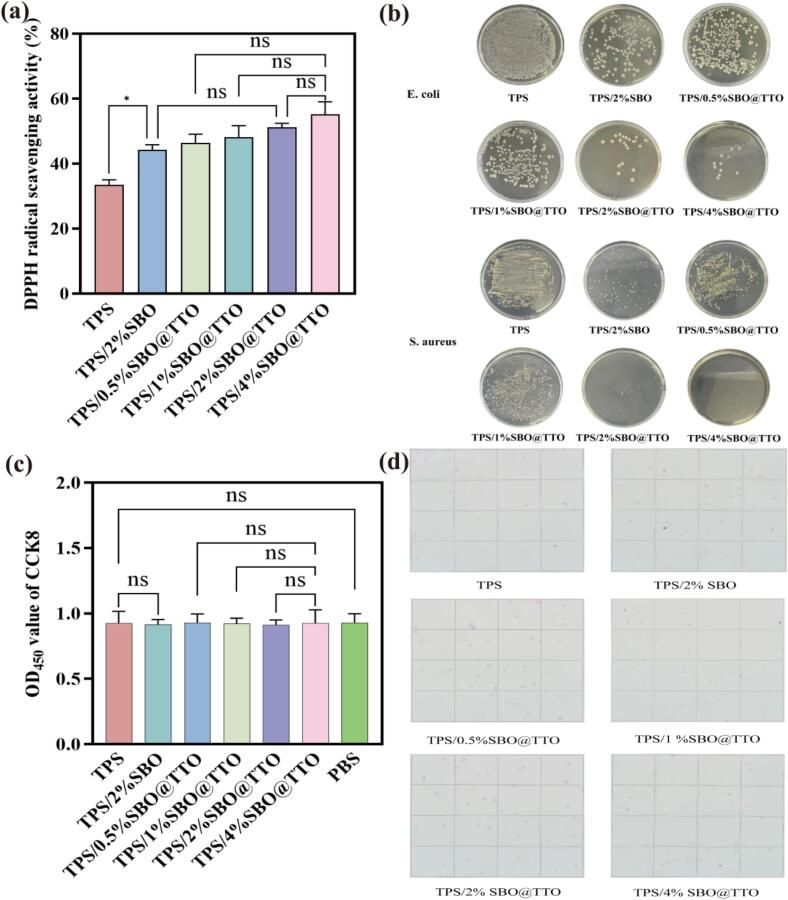
Activity and toxicity of the starch membranes, (a) antioxidant activity, (b) antibacterial activity, (c) cell viability assay, and (d) trypan blue staining. Asterisks indicate significant differences: *P < 0.05, **P < 0.01, ***P < 0.001. (For interpretation of the references to color in this figure legend, the reader is referred to the web version of this article.)

The antioxidant activities of the starch membranes are evaluated using the DPPH assay, as shown in Fig. 7(a). The *R*_DPPH_ value of the TPS membrane is 33.5 ± 1.5%, whereas that of the TPS/2%SBO membrane (44.1 ± 1.6%) is significantly higher (*P* < 0.05). The *R*_DPPH_ values of the TPS/0.5SBO@TTO, TPS/1%SBO@TTO, TPS/2%SBO@TTO, and TPS/4%SBO@TTO membranes are 44.6 ± 3.0%, 45.7 ± 2.8%, 48.9 ± 2.9%, and 55.2 ± 1.0%, respectively. Compared with the TPS membrane, the TPS/SBO@TTO membranes exhibit higher *R*_DPPH_ values. The triple antioxidant activity of the membrane arises from the synergistic action of BBR, OPCs, and TTO. The aromatic ring structure of BBR is an effective electron trap for free radicals, terminating oxidation chain reactions. The abundant hydroxyl groups on OPCs are oxidized to quinone structures, which interrupt the propagation of free radical chains. The phenolic constituents of TTO donate hydrogen atoms to deactivate free radicals (Li, Qin, et al., 2024). No significant differences are observed in the antioxidant activities of the TPS/SBO@TTO membranes. However, the antioxidant activity increases with increasing SBO@TTO content, which is attributed to the synergistic antioxidant mechanism of SBO and TTO.

The antibacterial activities of the starch membranes against *E. coli* and *S. aureus* are shown in Fig. 7(b). Compared to the TPS membrane, the inhibition rates of the TPS/2%SBO, TPS/0.5%SBO@TTO, TPS/1%SBO@TTO, TPS/2%SBO@TTO, and TPS/4%SBO@TTO membranes against *E. coli* are 88.1%, 66.1%, 78.7%, 98.7%, and 99.4%, respectively, and those against *S. aureus* are 95.5%, 41.1%, 49.0%, 99.2%, and 100%, respectively. The TPS/SBO@TTO membranes exhibit enhanced antibacterial activity against both strains as the SBO@TTO concentration increases. This could be attributed to the following reasons: (1) the active components in SBO compromise the integrity of bacterial cell membranes, leading to the leakage of intracellular contents and cell death; and (2) the terpene compounds in TTO interact with bacterial enzymes, inhibit metabolic processes, and induce oxidative stress (Xu et al., 2024; Wen et al., 2025; Li et al., 2024b).

The CCK-8 assay is used to quantitatively assess the metabolic activity of cell proliferation, as shown in Fig. 7(c). Compared to the control group, there is no marked increase in cell mortality across all starch membranes, and inter-group comparisons did not yield significant differences (*P* > 0.05). This indicates that the starch/SBO@TTO membranes exhibit satisfactory in vitro cytocompatibility with LO2 cells and show no evidence of eliciting cytotoxic responses under the experimental conditions used in this study (Zhu et al., 2024).

The toxicity of the starch membranes is verified using trypan blue exclusion staining, as shown in Fig. 7(d). The absence of dye uptake indicates that the cells retained their membrane integrity, whereas blue staining indicates cells death. Only small amounts of blue staining are observed in all samples, suggesting that most of the cells retain membrane integrity. These observations are consistent with the CCK-8 results, further supporting the satisfactory in vitro cytocompatibility of the starch membranes. Nevertheless, the results of the cytocompatibility assays do not substitute for formal safety evaluations. The potential migration of BBR, OPCs, and TTO from the membrane into food or food simulants must be quantified in future studies to assess compliance with regulatory safety standards for food-contact materials. This represents a critical direction for future investigation.

### 3.8 Release kinetics of tea tree oil from the starch membrane

**Fig. 8:**
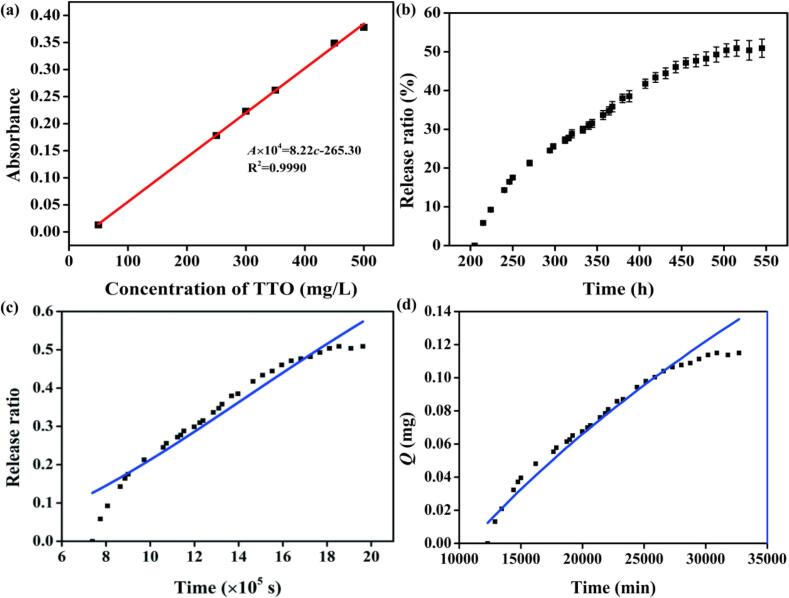
Release kinetics of TTO from the TPS/4%SBO@TTO membrane, (a) standard curve, (b) release kinetics, (c) Avrami model, and (d) Higuchi model.

The standard curve for TTO in Fig. 8(a) demonstrates a linear response described by the fitted equation *A* × 10^4^ = 8.22 × *c* – 265.30. The high *R*^2^ of 0.9990 confirms the robustness of the fit. This strong linearity validates the analytical method for accurate concentration measurements within the tested range.

The release kinetics of TTO from the TPS/4%SBO@TTO membrane are illustrated in Fig. 8(b). The release profile shows a typical biphasic pattern: an initial slow-release phase (0–205 h) with very low cumulative release, followed by a more rapid release phase (205–503 h) before approaching equilibrium. The initial slow release is attributed to the strong encapsulation of TTO within the SBO and effective retention by the starch matrix, which limits the rapid diffusion of the active compound within the matrix.

As shown in Fig. 8(c), the Avrami model yields the equation *R* = 1 – exp.(− (4.68 × 10^−7^ × *t*)^n^) with R^2^ = 0.9368. The fitted Avrami exponent is *n* = 0.85 (95% CI: 0.78–0.92), and the rate constant k is 4.68 × 10^−7^ s^−1^, indicating the sustained release behavior of TTO from the TPS/4%SBO@TTO membrane. The Higuchi model, depicted in Fig. 8(d), is described by the equation *Q* = 3.47 × 10^−3^ × (2.31 × 10^−2^ × *t*)^1/2^, with an *R*^2^ value of 0.9692. Compared with the Avrami model, the Higuchi model more accurately describes the release process, suggesting that the release of TTO from the TPS/4%SBO@TTO membrane is governed by a diffusion-controlled mechanism (Kumar et al., 2025). Diffusion-controlled release enables the membrane to sustain the release of the active ingredients in practical applications.

### 3.9 Preservation of cherry tomatoes using starch membranes

**Fig. 9:**
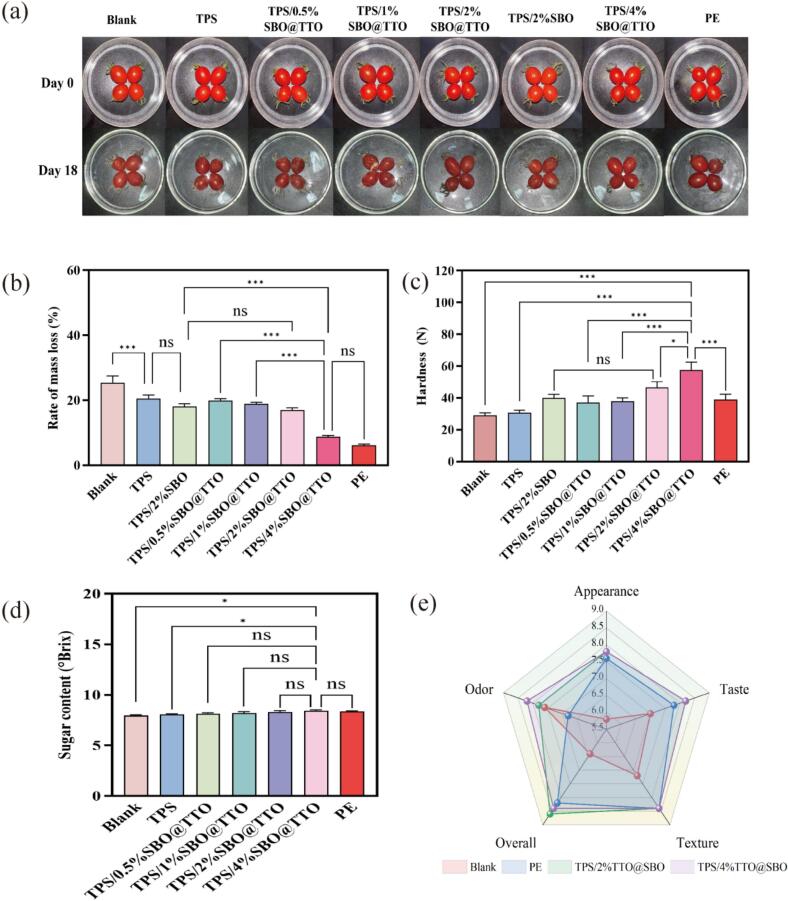
Quality of cherry tomatoes preserved by the starch membranes for 18 days, (a) appearance, (b) weight loss rate, (c) hardness, (d) sugar content, and (e) sensory evaluation. Asterisks indicate significant differences: *P < 0.05, **P < 0.01, ***P < 0.001.

The quality of cherry tomatoes preserved using the starch membranes for 18 days is shown in Fig. 9 and S4. As shown in Fig. 9(a), tomatoes stored in air or wrapped with commercial polyethylene (PE) membranes exhibit significant spoilage, whereas those packaged with TPS/SBO@TTO membranes maintained a markedly better appearance. This improvement is likely attributed to the strong antibacterial and antioxidant properties of SBO@TTO, as previously reported for these bioactive compounds (Xu et al., 2024; Wen et al., 2025; Li et al., 2024b).

As shown in Fig. 9(b), the weight loss of tomatoes packaged with TPS/SBO@TTO membranes is lower than that of tomatoes stored in air after 18 days. Moreover, the weight loss decreases with increasing SBO@TTO content, which is hypothesized to be attributed to the improved gas barrier properties and enhanced antioxidant activity of the starch/SBO@TTO membranes. Notably, the weight loss of tomatoes wrapped with TPS/4%SBO@TTO membrane is slightly higher than that of tomatoes wrapped with commercial PE membrane, although the difference is not significant (*P* > 0.05).

As depicted in Fig. 9(c), the hardness of tomatoes packaged with TPS/SBO@TTO membranes shows an inverse trend to the weight loss. The highest hardness is observed for tomatoes wrapped with the TPS/4%SBO@TTO membrane, which may be attributed to its superior gas barrier and antioxidant activity (Li et al., 2024a).

Fig. 9(d) shows that the sugar content of tomatoes wrapped with the TPS/4%SBO@TTO membrane (8.4 ± 0.1°Brix) is significantly higher than that of tomatoes stored in air (7.9 ± 0.1°Brix, P < 0.05) and higher than that of tomatoes wrapped with a commercial PE membrane (8.1 ± 0.1°Brix, P > 0.05). This superior sugar retention may be attributed to a reduced respiration rate and delayed senescence, although these parameters were not directly measured in the present study (Li et al., 2024a).

In Fig. S4(a), the *a* value, which reflects the red-green color balance (positive = red), is significantly higher for tomatoes wrapped with the TPS/4%SBO@TTO membrane than for those tomatoes wrapped with the commercial PE membrane, indicating that the membrane better maintained the characteristic red color of ripe cherry tomatoes. As shown in Fig. S4(b), after 18 days of storage, the pH of tomatoes wrapped with the TPS/4%SBO@TTO membrane (4.08 ± 0.04) is significantly higher than that of tomatoes stored in air (3.83 ± 0.04, P < 0.01), indicating that the membrane effectively retarded the acidification associated with fruit senescence and microbial spoilage.

These comprehensive quality assessments confirm that the TPS/4%SBO@TTO membrane effectively preserves cherry tomatoes by maintaining multiple quality attributes, including sugar content, pH, color, and sensory acceptability (Fig. 9(e)), through its synergistic antioxidant, antibacterial, and gas barrier properties. The observed preservation effects are hypothesized to be associated with a combination of factors. First, the known antioxidant activities of BBR, OPCs, and TTO, as extensively documented in the literature (Xu et al., 2024; Wen et al., 2025; Li et al., 2024b), are expected to help mitigate oxidative stress during fruit senescence. Second, the antibacterial properties of these compounds likely reduce microbial proliferation on the fruit surface. Third, the enhanced gas barrier properties of the composite membranes may contribute to reduced respiration rates and nutrient consumption (Wongphan et al., 2025). However, we acknowledge that the current study did not directly measure key biochemical markers of fruit senescence, including reactive oxygen species (ROS) levels, polyphenol oxidase (PPO) and peroxidase (POD) activities, malondialdehyde (MDA) content, lycopene degradation, or respiration rate. Therefore, the proposed molecular mechanisms remain speculative and hypothesis-generating rather than conclusively demonstrated. Direct confirmation of these pathways requires future investigations employing targeted biochemical assays.

## 4 Conclusions

This study demonstrates the successful fabrication of a smart and active starch-based membrane functionalized with SBO@TTO nanoparticles. The core SBO carrier was structurally characterized and was found to efficiently load TTO, following an adsorption process best described by the Langmuir isotherm and pseudo-first-order kinetics. The integration of SBO@TTO into the TPS matrix significantly enhanced its physicochemical properties. Key improvements include superior mechanical strength, enhanced hydrophobicity, and excellent barrier properties against water vapor and oxygen, which are attributed to strong hydrogen bonding and a more compact structure. In addition to these fundamental enhancements, the composite membranes exhibit advanced functionalities crucial for intelligent packaging. It provides strong UV protection, distinct pH-responsive colorimetric indicator, and potent concentration-dependent antioxidant and antibacterial effects. The TTO release profile was sustained and diffusion-controlled, ensuring prolonged activity. All formulations exhibit satisfactory in vitro cytocompatibility toward LO2 cells. The efficacy of the optimized TPS/4%SBO@TTO membrane is unequivocally validated in a practical food preservation test, where it significantly extends the shelf life of cherry tomatoes by minimizing spoilage, weight loss, and texture softening. Future work will address these limitations through comprehensive migration testing, expanded preservation studies across diverse food systems and storage conditions, and lifecycle environmental impact testing of the membranes. Despite these limitations, this study provides a solid foundation for developing starch-based active and intelligent packaging materials with triple antioxidant activity and pH-responsive indication capabilities.

## CRediT authorship contribution statement

**Kang Zhang:** Writing – original draft, Investigation, Funding acquisition. **Hefeng Zhu:** Software, Investigation. **Panliang Zhang:** Validation, Funding acquisition, Formal analysis. **Yixuan He:** Writing – review & editing, Methodology, Conceptualization. **Yixiu Wang:** Writing – review & editing, Funding acquisition.

## Declaration of competing interest

The authors declare that they have no known competing financial interests or personal relationships that could have appeared to influence the work reported in this paper.

## Data Availability

The authors are unable or have chosen not to specify which data has been used.
