# Supplementary material for: Starch-based intelligent indicator membranes with triple antioxidant activity for fruit preservation via self-assembled berberine-oligomeric proanthocyanidins nanoparticles encapsulating tea tree oil
Source: Food Chem X. 2026 Jul 20;38:104224. doi: 10.1016/j.fochx.2026.104224 (PMC13396897; doi:10.1016/j.fochx.2026.104224)
Supplement: Supplementary file 1 — Supplementary material [file mmc1.docx]

**Starch-based intelligent indicator membranes with triple antioxidant activity for fruit preservation via self-assembled berberine-oligomeric proanthocyanidins nanoparticles encapsulating tea tree oil**

Kang Zhang^a^, Hefeng Zhu^a^, Panliang Zhang^a^,Yixuan He^a*^, Yixiu Wang^b^

^a^ Department of Chemistry and Chemical Engineering, Hunan Institute of Science and Technology, Yueyang 414006, China; ^b^ Department of Hepatic Surgery, Fudan University Shanghai Cancer Center, Shanghai 200032, China.

***** Corresponding authors:

1. mail addresses: [hhh2500685410@163.com (Y. He)](mailto:18202888476@163.com;).


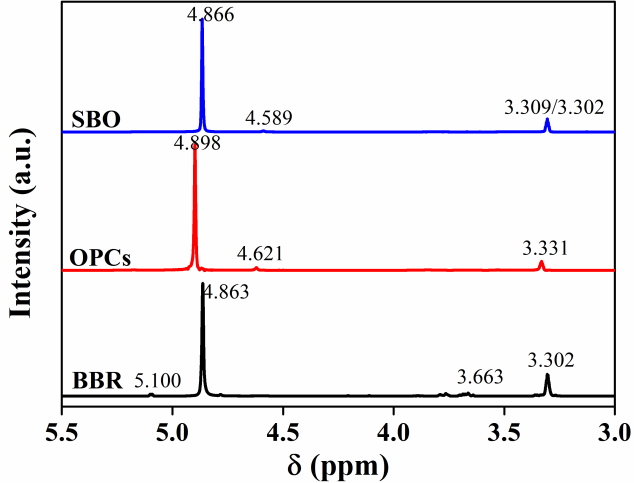


**Figure S1** ^1^H NMR spectra of the self-assembled nanoparticles and their corresponding raw materials.


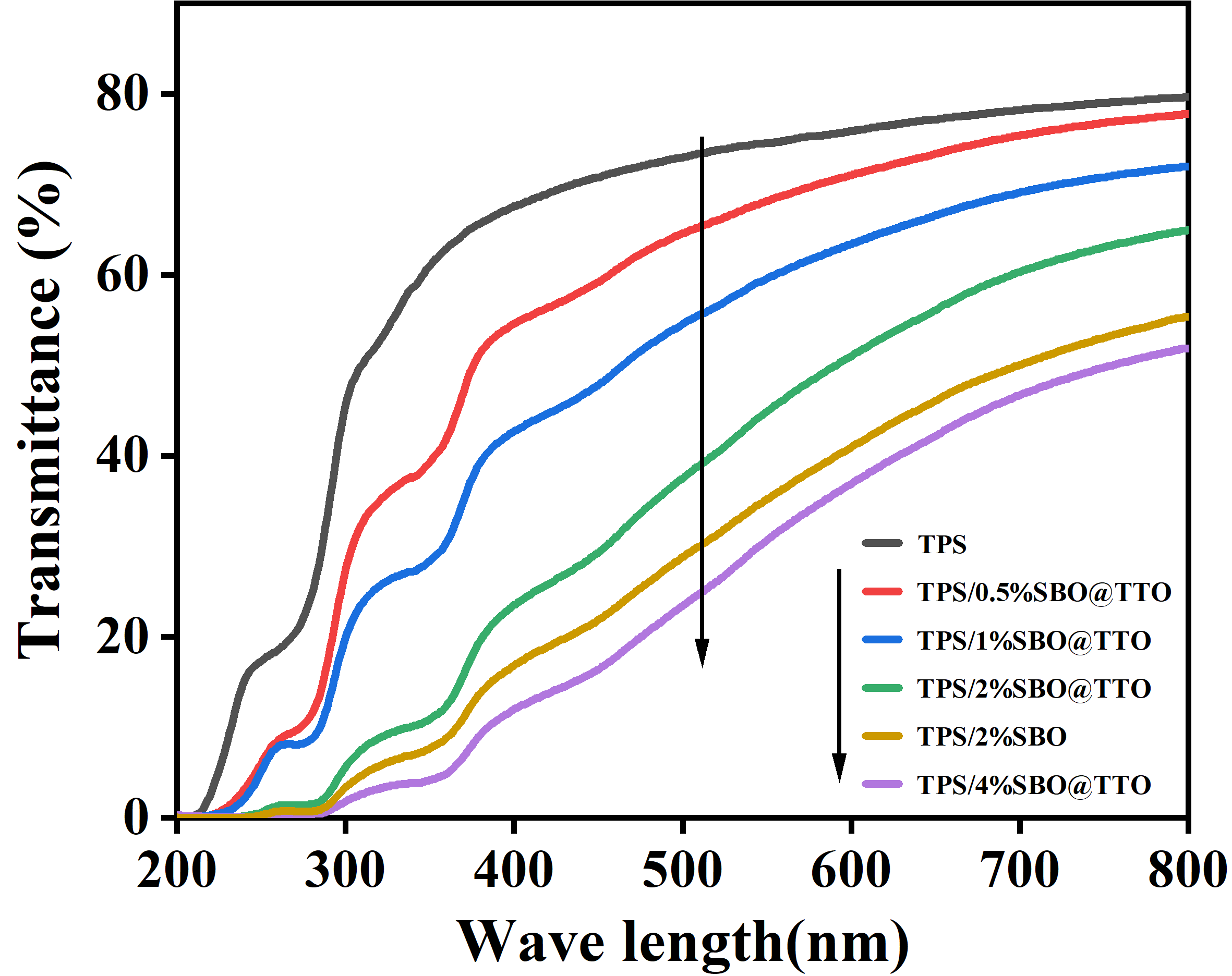


**Figure S2** UV-Vis curves of the starch membranes.


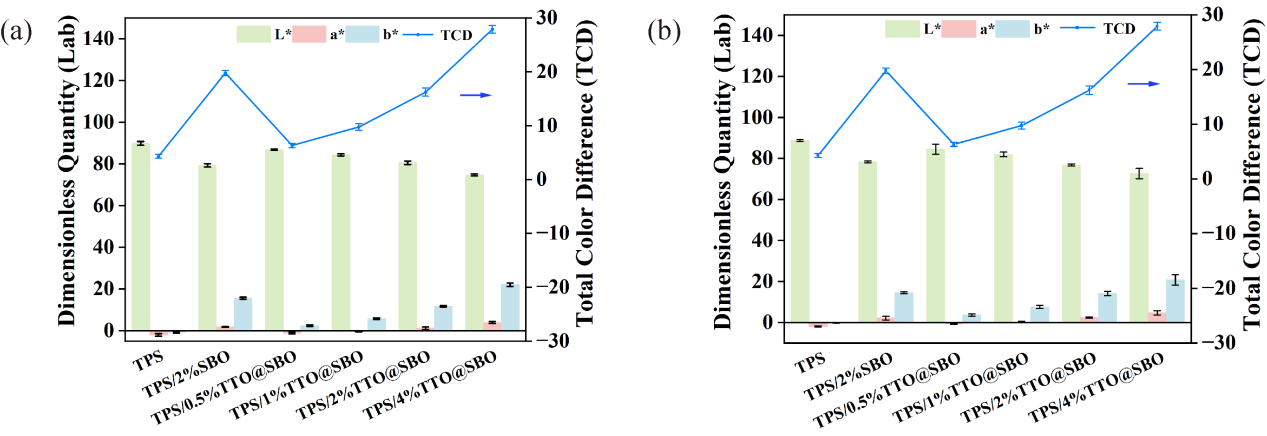


**Figure S3** Color parameters of the starch membranes before (a) and after the 18-day cherry tomato preservation experiment (b).


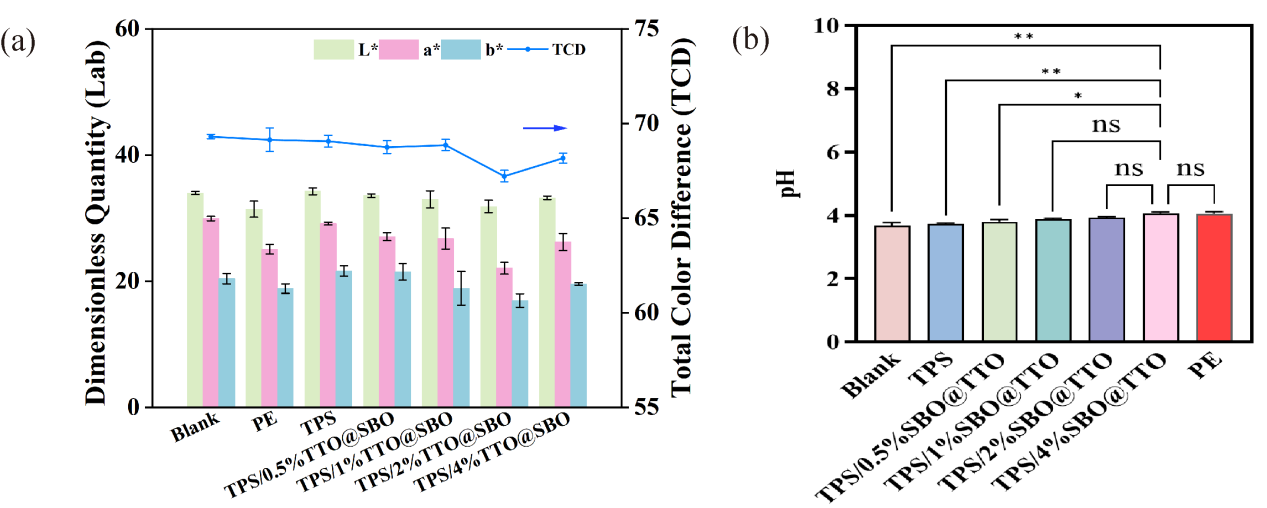


**Figure S4** Color parameters (a) and pH (b) of cherry tomatoes preserved by the starch membranes for 18 days.
